# Development of mental health first aid guidelines for panic attacks: a Delphi study

**DOI:** 10.1186/1471-244X-9-49

**Published:** 2009-08-10

**Authors:** Claire M Kelly, Anthony F Jorm, Betty A Kitchener

**Affiliations:** 1 ORYGEN Research Centre, University of Melbourne, Australia

## Abstract

**Background:**

Panic attacks are common, and while they are not life-threatening events, they can lead to the development of panic disorder and agoraphobia. Appropriate help at the time that a panic attack occurs may decrease the fear associated with the attack and reduce the risk of developing an anxiety disorder. However, few people have the knowledge and skills required to assist. Simple first aid guidelines may help members of the public to offer help to people who experience panic attacks.

**Methods:**

The Delphi method was used to reach consensus in a panel of experts. Experts included 50 professionals and 6 people who had experience of panic attacks and were active in mental health advocacy. Statements about how to assist someone who is having a panic attack were sourced through a systematic search of both professional and lay literature. These statements were rated for importance as first aid guidelines by the expert and consumer panels and guidelines were written using the items most consistently endorsed.

**Results:**

Of 144 statements presented to the panels, 27 were accepted. These statements were used to develop the guidelines appended to this paper.

**Conclusion:**

There are a number of actions which are considered to be useful for members of the public to do if they encounter someone who is having a panic attack. These guidelines will be useful in revision of curricula of mental health first aid programs. They can also be used by members of the public who want immediate information about how to assist someone who is experiencing panic attacks.

## Background

Panic attacks are common, with a US survey showing a lifetime prevalence of approximately 28% and 12-month prevalence of approximately 11% [1]. Panic attacks may lead to the development of panic disorder or agoraphobia which have prevalence rates in the range 1–5% [1-5]. Both disorders are associated with a high degree of impairment and co-morbidity with other psychiatric disorders [6-9].

For someone who has experienced a panic attack, there are a number of factors which increase the risk of developing panic disorder or agoraphobia. Catastrophic misinterpretations (for example, fear that one is having a heart attack or other medical emergency) in relation to panic symptoms predict the onset of panic disorder and agoraphobia [10]. Severity of panic attacks, as measured by number of physical symptoms, as well as number of catastrophic cognitions, appears to increase the risk of developing agoraphobia among adolescents [11]. The presence of a specific cognition, 'fear of going crazy or losing control', during the first panic attack predicts the onset of agoraphobia rather than panic disorder [3]. Pre-existing high trait anxiety and the presence of other psychiatric illnesses, particularly depression, predict the onset of either panic disorder or agoraphobia among those who have had a first episode of panic [12]. A focus on the possibility of future panic attacks, hyper-vigilance about physical symptoms and catastrophic cognitions increase the risk of developing panic disorder or agoraphobia [10].

An appropriate response by a member of the public, whether a friend, family member, co-worker or other person, may in some cases decrease the likelihood that an individual who experiences a panic attack will go on to be hyper-vigilant about physical symptoms or fear future panic attacks, thus decreasing the likelihood of developing a panic-related psychiatric disorder.

In this paper, we aim to improve one particular approach to public education – training of members of the public in how to give first aid to someone who is experiencing a panic attack. One existing approach of this sort is the Mental Health First Aid training program [13]. Mental Health First Aid training [14] was developed to train members of the public to assist others who are developing a mental disorder or in a mental health crisis situation. First aid givers can be almost anyone, however, they are most likely to be friends, family members or colleagues, simply because they are the people most likely to be present at the time first aid is needed.

When the program was first in development, the authors used evidence-based information wherever possible, but very little research was found about how members of the public, with no clinical training, could assist in these situations. Where no evidence was available, the authors informally sought the opinions of clinical experts.

In order for these approaches to be effective, it needs to be ensured that the first aid strategies taught are likely to be helpful. Because controlled trials of individual components of first aid strategies are not feasible, an alternative is to use expert consensus to develop a set of guidelines on strategies that are most likely to work. Such guidelines can be used directly as a source of advice by members of the public and they can inform the content of first aid training courses. The aim of this project was to develop such guidelines. These guidelines needed to focus on the immediate response to a discrete panic attack, and not on diagnosing or treating a panic-related psychiatric condition. The first aid giver's role would be to assist the person until the crisis has passed or the person has chosen to seek appropriate professional help.

We chose the Delphi method, a technique used for reaching consensus in a group of experts or across expert groups. Our aim was to get consensus within and between panels of professionals, carers and consumers, so that the guidelines would be respectful of the needs of all three groups. This method is relatively inexpensive and simple to conduct, and can be done on the Internet, making it possible to include participants from English-speaking countries across the world without lengthy postal delays. The Delphi methodology has been used in health research in the past, mainly to reach consensus amongst medical practitioners, but also with consumers of health services in some settings [15,16]. We have also successfully used this method to develop mental health first aid guidelines for depression, psychosis, eating disorders, suicidal thoughts and behaviours, traumatic events, and non-suicidal self-injury using panels of professionals, consumers and carers [17-21]. No research using the Delphi methodology to determine consensus on panic first aid guidelines has been conducted previously.

## Methods

**Figure 1:**
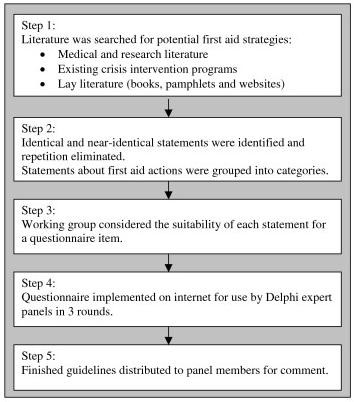
**Stages in guideline development**.

This study had two phases: a literature search and questionnaire development, and the Delphi process. Please see Figure 1 for a summary of the steps.

### Literature search

The aim of the literature search was to cover the full domain of potentially helpful actions to assist someone who is experiencing a panic attack. It was not a literature review and did not include literature outside of the scope of first aid. The focus for the search was to find statements which instruct the reader on how to respond at the time of the attack, and how and when to recommend professional help to someone who has experienced a panic attack. The literature search was conducted across three domains: (1) the medical and research literature, (2) the content of existing crisis intervention guidelines and relevant courses for the public, and (3) lay literature. The lay literature included books written for the general public, particularly consumers' and carers' guides, websites and pamphlets.

The medical and research literature was accessed through searches of PsycInfo and PubMed. The search term was 'panic attack AND intervention OR (first aid)' and all records for the 20 years leading to the search date were reviewed. Papers were excluded first on the basis of their titles and then on the basis of their abstracts.

Papers which described interventions to decrease the severity or duration of a panic attack, or offered suggestions about when to recommend professional help, were reviewed, giving a total of 37 papers. Most of the advice given in these papers was very clinically oriented, or required extensive training to be applicable. For example, some papers described clinical interventions in which a panic attack was induced in vivo and then de-escalated. A small number of papers did include brief advice and simple intervention instructions. Statements were drawn from 12 of the 37 relevant records. All statements felt to be simple enough for lay people to use were included.

To find appropriate websites, we used the search engines Google [22], Google Australia [23], and Google UK [24] using three sets of search terms; 'panic attack' and 'self help', 'panic attack' and 'first aid', and 'panic attack' and (care or carer or caring). The first 50 websites listed by each were reviewed; beyond the first 50 websites, most new records were abstracts from journal papers. Since most websites were listed by more than one search engine, and were retrieved for more than one of the search terms, 178 websites were reviewed. The websites were read thoroughly, once again looking for statements which suggested a potential first aid action (what the first aid giver should do) or relevant awareness statement (what the first aid giver should know). Any external links to other websites were followed and the same process applied to each of them.

The fifty most popular books on the Amazon [25] website which listed the word 'panic' in the title or keywords were selected. This site was chosen because of its extensive coverage of books in and out of print, including works about mental health aimed at the public. Books which were autobiographical in nature and clinical manuals were excluded. The remaining books were read to find useful statements. The majority of these were carers' guides, which do contain advice relevant for first aid, but focussed on general caring for a mentally ill family member.

Any relevant pamphlets were sought and read, and statements were taken from these as well.

Only one training course for members of the public was found to be relevant, as most training in critical incident response is designed for professional responders such as paramedics and the police. Material from the Mental Health First Aid Program [14] was reviewed and statements drawn from it.

### Questionnaire development

The questionnaire was developed by first grouping statements into the following categories: general intervention principles; de-escalating a panic attack; slowing down a person's breathing; things to say during a panic attack; professional help during a panic attack; alternative approaches to stopping a panic attack; seeking professional help, and self-help strategies.

Similar or near-identical statements were frequently derived from multiple sources, and they were not repeated in the questionnaire. A working group comprised of the authors of this paper and colleagues working on similar projects convened at each stage of the process to discuss each item in the questionnaire. The role of the working group was to ensure that the questionnaire did not include ambiguity, repetition, items containing more than one idea or other problems which might impede comprehension. The wording was carefully designed to be as clear, unambiguous and action-oriented as possible. All participants answered the questionnaire via the Internet, using an online survey website, Surveymonkey [26].

### The Delphi process

The aim was to recruit participants into one of three panels: professionals (clinicians and researchers), consumers (people who had experienced panic attacks in the past) and carers. The professional panel had 50 experts, the consumer panel 6, but no carers could be recruited. All panel members were from developed English speaking countries (Australia, New Zealand, The United States, Canada, Ireland, England, and the United Kingdom). Participants were recruited in a number of ways. Professionals recruited were those who had publications in the areas of panic disorder or agoraphobia or experience in treating these patients. When letters were sent to professionals asking them to be involved, they were also invited to nominate any colleagues who they felt would be appropriate panel members. Those active in clinical practice were also asked to consider any former patients who might be willing to be involved.

The 50 professional participants belonged to the following (sometimes multiple) groups: 44 academics (researchers, lecturers and professors), 23 clinical psychologists, 21 medical doctors of whom 12 were psychiatrists, 1 nurse (also an academic), 1 clinical social worker and 1 drug and alcohol counsellor working with anxiety patients with tranquilliser addiction.

Consumers were recruited from advocacy organisations and referral by clinicians. They were also identified if they had written websites offering support and information to other consumers. Consumers were difficult to recruit for this study. All six consumers were working in some form of advocacy role. In addition, 1 was an academic researcher, 1 was the convener of a mutual help group, and 1 was a clinical psychologist who chose to participate as a consumer.

Many attempts were made to recruit carers from carers' support organisations and informal sources, but no carers chose to participate in this study. It may be that many carers for people with panic related conditions do not identify themselves as such. They may be a group that is less inclined to be involved with carers' organisations than those who are carers for people with schizophrenia, depression, or eating disorders [19-21]. Similar difficulties were found recruiting carers for people who have been suicidal or engaged in non-suicidal self-injury [17,18].

Three rounds of questionnaires were distributed as follows, with each statement being rated up to two times. In Round 1 the questionnaire, derived from the process described above, was given to the panel members. The questionnaire included space after each of the sections to add any suggestions for new statements that panel members felt should be included.

In each round of the study, the usefulness of each statement for inclusion in the mental health first aid guidelines was rated as 'essential', 'important', 'don't know or depends', 'unimportant', or 'should not be included'. The options 'don't know and depends' were collapsed into one point on the scale because operationally, they are the same response. Most of the statements were, very reasonably, noted to be useful in some cases and not others, meaning they could not be generalised in guidelines, which is also true of statements participants did not feel confident to rate.

The suggestions made by the panel members in Round 1 were reviewed by the working group and used to construct new items for the Round 2. Suggestions were accepted and added to Round 2 if they represented a truly new idea, could be interpreted unambiguously by the working group, and were actions. Suggestions were rejected if they were near-duplicates of items in the questionnaire, if they were too specific (for example, 'focus on the guided meditation imagery negotiated between myself and my psychologist'), too general ('just be there'), or were more appropriate to therapy than first aid ('remember to avoid using safety behaviours'). Unexpectedly, in Round 1, no items describing techniques to control breathing were endorsed by the professional panel, although many were endorsed by the consumer panel. The working group chose to add any new item about breathing techniques suggested by panel members, in spite of some being close to duplicates of Round 1 items, in case one was felt to be acceptable to the professional panel.

Items rated as 'essential' or 'important' by 80% or more of both the professional panel and the consumer panel were accepted for inclusion in the guidelines. If they were endorsed by 80% or more of one of the panels, or by 70–80% of both panels, they were re-rated in the subsequent round. Items which met neither condition were rejected. Before Round 2 and 3 of the study, each participant was sent a summary of the results of the previous round, listing which items had been accepted, which had been rejected, and which were to be re-rated. When an item was to be re-rated by the panellists, they were provided with their own response and a table outlining how many people in each group had endorsed the item. They were told that they did not have to change their responses when re-rating an item, but that if they wished to, they would have the opportunity to do so.

## Results

Table 1 shows the continuity of participation across the three rounds.

**Table 1:** Study participation in each round

Panel	Round 1	Round 2	Round 3
Consumers	6	5	3
Professionals	50	44	35

**Figure 2:**
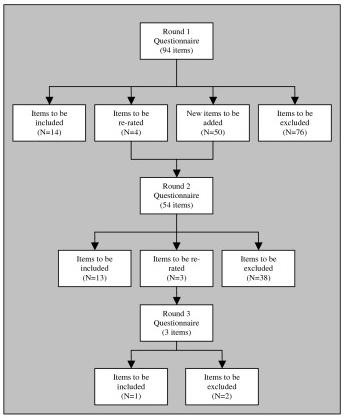
**Items accepted, rejected and re-rated at each round**.

Figure 2 shows the rates of inclusion, exclusion, and re-rating of the items in each round of the questionnaire. From a total of 144 items, 27 were eventually included in the guidelines. (See Table 2 for a categorised list of included items.)

**Table 2:** Statements accepted as mental health first aid guidelines

**General intervention principles:**	**Round:**
The first aider should identify themselves if they are not known to the person.	1
The first aider should explain to the person that they are experiencing a panic attack.	1
The first aider should speak to the person in a reassuring but firm manner.	1
The first aider should remain calm and avoid becoming caught up in the panic.	1
The first aider should speak clearly and slowly.	1
The first aider should use short, clear sentences.	1
The first aider should be patient with the person.	1
The first aider should acknowledge that the person's terror feels very real to them.	1
The first aider should reassure the person that a panic attack will rarely last more than ten minutes.	2
The first aider should know the symptoms of a panic attack.	2
The first aider should ask the person if they know what is happening.	2
If the person says that they are having a panic attack, the first aider should ask them if they need any kind of help, and give it to them.	2
The first aider should ask the person if they have ever had a panic attack before.	3
	
**De-escalating a panic attack**	
	
*No items endorsed.*	
	
**Slowing down the person's breathing:**	
	
*No items endorsed.*	
	
**Things a first aider should say during a panic attack:**	
Rather than making assumptions about what the person needs, the first aider should ask them directly.	1
The first aider should reassure the person that a panic attack, while very frightening, is not life threatening.	1
The first aider should reassure the person that a panic attack, while very frightening, is not dangerous.	1
The first aider should not belittle the person's experience.	1
The first aider should reassure the person that they are safe.	2
The first aider should reassure the person that the symptoms will pass.	2
	
**Professional help in an emergency:**	
	
If the person loses consciousness, the first aider should apply regular first aid principles (check for breathing and pulse).	2
If the person loses consciousness, the first aider should call an ambulance.	2
	
**Alternative approaches to stopping a panic attack:**	
	
*No items endorsed.*	
	
**Seeking professional help**	
	
The first aider should assure the person that effective treatments are available for panic disorder.	1
The first aider should be aware of the range of professional help available for panic attacks.	2
The first aider should tell the person that if the panic attacks recur, and are causing them distress, they should speak to an appropriate health professional.	2
The first aider should assure the person that panic attacks and panic disorder can be effectively treated.	2
The first aider should ask the person if they know where they can seek help and advice about panic attacks. If the person doesn't know, the first aider should offer some suggestions.	2
	
**Self-help strategies:**	
	
After the panic attack has stopped, the first aider should explain to the person where they can get more information about panic attacks.	2

### Writing the Guidelines

It was important to the research team to avoid making the guidelines read like a list of 'dos' and 'don'ts'. The accepted items were incorporated into a plain language document. To illustrate, consider the following statements:

1. The first aider should reassure the person that a panic attack, while very frightening, is not life threatening.

2. The first aider should reassure the person that a panic attack, while very frightening, is not dangerous.

3. The first aider should not belittle the person's experience.

4. The first aider should reassure the person that they are safe.

5. The first aider should reassure the person that the symptoms will pass.

These statements were incorporated to make the following paragraph:

Do not belittle the person's experience. Acknowledge that the terror feels very real, but reassure them that a panic attack, while very frightening, is not life threatening or dangerous.

When the guidelines were in draft form, they were sent to all the panel members for feedback. Only feedback related to readability and structure was sought and incorporated. The guidelines are appended to this article and can be freely distributed (see additional file 1).

## Discussion

Significant differences between the consumer and professional panels were evident in this study. In particular, de-escalating panic attacks through breathing techniques was seen as important by the consumer panel, but not by the professional panel. In total, 23 items relating to breathing were rated by the panels. Of these, 6 were endorsed by the consumer panel (5 in Round 1), but none were endorsed by the professional panel. This was very interesting to the research team, as much of the lay literature about panic attacks emphasised the importance of controlled breathing.

In Round 1, many of the professional respondents stated that hyperventilation and other breathing difficulties were not common in panic attacks. We thought this may be the reason for the low level of endorsement, and so, in Round 2, we altered the wording of the breathing-related items so that they each read "If the person is having trouble with their breathing, the first aider should..." followed by the breathing technique. This made no difference to the ratings. Professional respondents also stated that a reliance on controlled breathing could cause difficulties later on for people who sought help for their panic attacks, as these could become 'safety behaviours' which interfered with real progress in coping with panic. Alternatively, it may be that the professionals simply didn't think that breathing techniques were important enough to be listed in the guidelines. Our cut-off for inclusion was 80% – a less conservative cut-off would have seen more items included. Only the most essential action are included in the guidelines.

Of great concern to many respondents was the idea that a first aid giver could distinguish a panic attack from a heart attack or other serious medical problem. The attached guidelines do not encourage first aid givers to make any such distinction; rather, they are conservative, encouraging that first aid be given for a panic attack only if the person has experienced a panic attack before, believes they are experiencing one now, and does not have symptoms more indicative of a serious medical problem.

In order to be able to effectively apply the recommended panic first aid strategies, individuals will need to have either recently or consistently been exposed to the messages in the guidelines document. This is a criticism which could be made of all first aid approaches, and is a significant concern. Physical first aid courses are usually accredited and need to be renewed on a regular basis, and ultimately mental health first aid should be similarly regimented.

Finally, there were issues in regards to the items about seeking professional help after a panic attack. Items about encouraging any person who had experienced a panic attack to seek professional help of any kind were not highly endorsed, although it was felt that first aid givers should tell the person that effective treatments are available. Certainly, a large number of people experience a panic attack at some stage in their lives and do not go on to develop panic disorder or agoraphobia. The most significant item included in the guidelines in regards to professional help was one stating that, if the person continued to have panic attacks and felt distressed by them, they should seek help from an appropriate professional. This item should assist those who are most at risk of developing panic disorder or agoraphobia to get professional help early.

## Limitations

A significant limitation of this study is the small number of consumer panel members and the lack of a carers' panel. Ideally, this study would have involved approximately equal numbers of professional, consumer and carer panellists, but recruiting consumers was very difficult and recruiting carers even more so. As only six consumers were involved in the development of these guidelines, it is possible that their opinions are not representative of people with panic disorder and agoraphobia more generally. This project should be conducted again at some stage in the future with a carers panel and a larger consumer panel.

It is important as well to reiterate that all panellists were recruited from developed English-speaking countries, so that the guidelines may not be generalisable to other countries or to minority cultures within those countries. Furthermore, these guidelines cannot stand alone, as they do not address the underlying psychological distress or mental illness which may predispose an individual to begin experiencing panic attacks or go on to suffer from a panic-related psychiatric illness. Other guidelines in this series may be useful in this regard [17-21].

## Conclusion

We have succeeded in developing guidelines for first aid for panic attacks which are acceptable to both professionals and people who have experienced panic attacks. Where the guidelines are used as the basis for first aid training, efforts need to be made to evaluate their impact on the first aid givers' helping behaviours and on the recipients of the first aid, as far as this is possible.

## Competing interests

The authors declare that they have no competing interests.

## Authors' contributions

CMK and AFJ prepared the manuscript. All authors reviewed the manuscript. AFJ and BAK developed the methodology. CMK did the literature searches and wrote the first draft of the questionnaire. All authors contributed to the development of later versions of the questionnaire. CMK wrote the attached guidelines. All authors reviewed and suggested improvements to the guidelines. All authors read and approved the final manuscript.

## Pre-publication history

The pre-publication history for this paper can be accessed here:



## Supplementary Material

Additional file 1 **First aid guidelines for panic attacks**. This file may be distributed freely, with the authorship and copyright details intact. Please do not alter the text or remove the authorship and copyright details. Click here for file
