# Supplementary material for: Development of mental health first aid guidelines for panic attacks: a Delphi study
Source: BMC Psychiatry. 2009 Aug 10;9:49. doi: 10.1186/1471-244X-9-49 (PMC2739201; doi:10.1186/1471-244X-9-49)
Supplement: Additional file 1 — First aid guidelines for panic attacks. This file may be distributed freely, with the authorship and copyright details intact. Please do not alter the text or remove the authorship and copyright details. [file 1471-244X-9-49-S1.pdf]

MENTAL HEALTH FIRST AID

PANIC ATTACKS

FIRST AID GUIDELINES

What is a panic attack?

A panic attack is a distinct episode of high anxiety, with fear or discomfort, which develops abruptly and has its peak within 10 minutes. During the attack, several of the following symptoms are present.

- Palpitations, pounding heart, or rapid heart rate
- Sweating
- Trembling and shaking
- Shortness of breath, sensations of choking or smothering
- Chest pain or discomfort
- Abdominal distress or nausea
- Dizziness, light-headedness, feeling faint or unsteady
- Feelings of unreality (derealisation), or being detached from oneself
- Fears of losing control or going crazy
- Fear of dying
- Numbness or tingling
- Chills or hot flushes

Adapted from DSM-IV-TR, APA 2000

The MHFA Training & Research Program

Orygen Youth Health Research Centre
Department of Psychiatry
The University of Melbourne
AUSTRALIA

www.mhfa.com.au

PANIC ATTACKS

FIRST AID GUIDELINES

MENTAL HEALTH FIRST AID

What should I do if I think someone is having a panic attack?

If someone is experiencing the above symptoms and you suspect that they are having a panic attack, you should first ask them if they know what is happening and whether they have ever had a panic attack before. If they say that they have had panic attacks before, and believe that they are having one now, ask them if they need any kind of help, and give it to them. If you are helping someone you do not know, introduce yourself.

What if I am uncertain whether the person is really having a panic attack, and not something more serious like a heart attack?

The symptoms of a panic sometimes resemble the symptoms of a heart attack or other medical problem. It is not possible to be totally sure that a person is having a panic attack. Only a medical professional can tell if it is something more serious. If the person has not had a panic attack before, and doesn't think they are having one now, you should follow physical first aid guidelines.

Ask the person, or check to see, if they are wearing a medical alert bracelet or necklace. If they are, follow the instructions on the alert or seek medical assistance.

If the person loses consciousness, apply physical first aid principles. Check for breathing and pulse, and call an ambulance.

What should I say and do if I know the person is having a panic attack?

Reassure the person that they are experiencing a panic attack. It is important that you remain calm and that you do not start to panic yourself. Speak to the person in a reassuring but firm manner, and be patient. Speak clearly and slowly and use short, clear sentences.

Rather than making assumptions about what the person needs, ask them directly what they think might help.

Do not belittle the person's experience. Acknowledge that the terror feels very real, but reassure them that a panic attack, while very frightening, is not life threatening or dangerous. Reassure them that they are safe and that the symptoms will pass.

What should I say and do when the panic attack has ended?

After the panic attack has subsided, ask the person if they know where they can get information about panic attacks. If they don't know, offer some suggestions.

Tell the person that if the panic attacks recur, and are causing them distress, they should speak to an appropriate health professional. You should be aware of the range of professional help available for panic attacks in your community. Reassure the person that there are effective treatments available for panic attacks and panic disorder.

Panic attacks, panic disorder and agoraphobia

A panic attack is not a mental disorder. In fact, more than one in five people experience one or more panic attacks in their lifetime[1], but few go on to develop panic disorder or agoraphobia (anxiety disorders related to panic attacks).

Criteria for panic disorder[2]

- Recurrent, unexpected panic attacks

AND, for at least one month:

- worry or concern about possible future panic attacks;
- worry or concern about the possible consequences of panic attacks, such as a fear of losing control or having a heart attack;
- or a significant change in behaviour related to the panic attacks.

Criteria for agoraphobia[2]

- Anxiety about places or situations where the individual fears they may have a panic attack. The focus of the anxiety is that it will be difficult or embarrassing to get away from the place if a panic attack occurs, or that there will be no one present who can help.

AND:

- Avoidance of the places or situations which are the focus of the anxiety.

Some individuals avoid only a few places or situations (such as shopping centres, driving, or crowded places) and others may find it difficult to leave their homes.

Some people may develop panic disorder or agoraphobia after only a few panic attacks, while others may experience many panic attacks without developing either of these disorders.

1. Kessler RC et. al. *Arch Gen Psychiatry 2006, 63:415-424*

2. Adapted from DSM-IV-TR, APA 2000

MENTAL HEALTH FIRST AID

PANIC ATTACKS

FIRST AID GUIDELINES

Purpose of these Guidelines

These guidelines are designed to help members of the public to provide first aid to someone who is having a panic attack. The role of the first aider is to assist the person until appropriate professional help is received or the crisis resolves.

Development of these Guidelines

The following guidelines are based on the expert opinions of a panel of mental health consumers and clinicians from Australia, New Zealand, the UK, the USA and Canada about how to help someone who is having a panic attack. Details of the methodology can be found in: (…)

How to use these Guidelines

These guidelines are a general set of recommendations about how you can help someone who is having a panic attack. Each individual is unique and it is important to tailor your support to that person's needs. These recommendations therefore may not be appropriate for every person who experiences a panic attack.

Also, the guidelines are designed to be suitable for providing first aid in developed English-speaking countries. They may not be suitable for other cultural groups or for countries with different health systems.

Although these guidelines are copyright, they can be freely reproduced for non-profit purposes provided the source is acknowledged.

Please cite these guidelines as follows:
Mental Health First Aid Training and Research Program. *Panic attacks: first aid guidelines.* Melbourne: Orygen Youth Health Research Centre, University of Melbourne; 2008.

Enquiries should be sent to:

Professor Tony Jorm, Orygen Youth Health Research Centre
Locked Bag 10, Parkville VIC 3052 Australia
email: ajorm@unimelb.edu.au

All MHFA guidelines can be downloaded from **www.mhfa.com.au**

8185_08 ORYGENKJ
